# An Environmentally Friendly Method for Testing Photocatalytic Inactivation of Cyanobacterial Propagation on a Hybrid Ag-TiO_2_ Photocatalyst under Solar Illumination

**DOI:** 10.3390/ijerph121215023

**Published:** 2015-12-11

**Authors:** Shu-Yu Chang, Winn-Jung Huang, Ben-Ren Lu, Guor-Cheng Fang, Yeah Chen, Hsiu-Lin Chen, Ming-Chin Chang, Cheng-Feng Hsu

**Affiliations:** 1 Kuang-Tien General Hospital, No. 117, Satien Road, Shalu District, Taichung 43303, Taiwan; hollyspirit31@yahoo.com.tw; 2 Department of Safety, Health and Environmental Engineering, Hungkuang University, No. 1018 Sec. 6, Taiwan Boulevard, Shalu District, Taichung 43302, Taiwan; gcfang@sunrise.hk.edu.tw (G.-C.F.); bluecrystalprotein@gmail.com (Y.C.); hsiulin@sunrise.hk.edu.tw (H.-L.C.); chang@sunrise.hk.edu.tw (M.-C.C.); yetihsu@yahoo.com.tw (C.-F.H.); 3 Department of Electronics and Communication Engineering, Peking University, No. 5, Zhuangyuan Road, Binhu District, Wuxi 214125, China; a22462863@yahoo.com.tw

**Keywords:** photocatalysis, silver, titanium dioxide, cyanobacteria, extracellular polymeric substances, disinfection by-products

## Abstract

Cyanobacteria were inactivated under sunlight using mixed phase silver (Ag) and deposited titanium dioxide (TiO_2_) coated on the surface of diatomite (DM) as a hybrid photocatalyst (Ag-TiO_2_/DM). The endpoints of dose-response experiments were chlorophyll a, photosynthetic efficiency, and flow cytometry measurements. *In vitro* experiments revealed that axenic cultures of planktonic cyanobacteria lost their photosynthetic activity following photocatalyzed exposure to sunlight for more than 24 h. Nearly 92% of *Microcystis aeruginosa* cells lost their photosynthetic activity, and their cell morphology was severely damaged within 24 h of the reaction. Preliminary carbon-14 (^14^CO_3_^−2^) results suggest that the complete inactivation of cyanobacteria arises from damage to cell wall components (peroxidation). A small concomitant increase in cell wall disorder and a consequent decrease in cell wall functional groups increase the cell wall fluidity prior to cell lysis. A high dosage of Ag-TiO_2_/DM during photocatalysis increased the concentration of extracellular polymeric substances (EPSs) in the *Microcystis aeruginosa* suspension by up to approximately 260%. However, photocatalytic treatment had a small effect on the disinfection by-product (DBP) precursor, as revealed by only a slight increase in the formation of trihalomethanes (THMs) and haloacetic acids (HAAs).

## 1. Introduction

Cyanobacteria are present in drinking water reservoirs worldwide and are a serious issue of great concern for drinking water authorities. The potential health hazards of toxin-producing cyanobacteria in drinking water supplies are well known [1]. The presence of cyanobacterial blooms can generate a wide range of toxins, including cyclic peptide hepatotoxins and alkaloid neurotoxins. The potential presence of cyanobacteria and their metabolites in water has led to the upgrading of drinking water treatment facilities with the addition of chemical oxidation, adsorption, and photocatalytic processes. The effectiveness of photocatalysis in disinfecting water has been demonstrated by its destructive effects on a wide range of microorganisms, including bacteria [2,3,4,5,6], viruses [7,8,9,10], fungi [11,12,13,14,15], and protozoa [16,17,18]. Numerous investigations have studied the role of experimental variables on the response of microorganisms to photocatalytic treatment [19,20,21,22,23]. These investigations have studied the effects of aeration, pH, the chemical nature of the bacterial suspension medium, the type and concentration of the photocatalyst, the light intensity, and the treatment time. While these experimental parameters have been shown to influence microbial responses to photoinactivation, the nature of the target organism must also be considered. Some investigations have established that microorganisms with more complex cell walls are more resistant to photocatalytic treatment, and the resistance of microorganisms to photocatalytic treatment reportedly follows the order of protozoa > bacterial spores > mycobacteria > cyanobacteria > viruses > fungi > bacteria [23].

TiO_2_ photocatalysts have been found to kill cancer cells, bacteria, viruses, and algae under UV illumination [24,25,26,27,28]. The antibacterial properties of TiO_2_ photocatalysis arise from the generation of reactive oxygen species, of which hydroxyl radicals regard as the most important. The accepted sequence of events that occurs when microorganisms undergo TiO_2_ photocatalytic attack begins with cell wall damage, followed by cytoplasmic membrane damage that results in a direct intracellular attack [29]. As a photocatalyst, TiO_2_ is activated by UV light wavelengths at or below 387 nm, and only a small proportion of the Sun’s energy is therefore utilized in oxidative transformations. Furthermore, several studies of the development of mixed-phase titania (anatase and rutile phase) have been performed to promote charge carrier generation/separation [30,31]. Generally, loading of noble metal co-catalysts such as Pt, Au, and Ag, *etc.*, enables accelerating liquid solid photocatalytic reactions [32,33]. The optimal deposition of metals such as silver (Ag) improves the transfer of the photogenerated charge carriers between the semiconductor and the metal, enabling separation of the electrons and holes [34,35,36]. Seery *et al.* reported that Ag doping in TiO_2_ is expected to show various effects on its photocatalytic activity by the following mechanisms [37,38]: (1) it may enhance the electron-hole separation by acting as electron traps; (2) it can extend the light absorption into the visible range, enhance surface electron excitation by plasmon resonances excited by visible light; and (3) modifivcation of the surface properties of photocatalysts. In various studies, the improvement in photocatalytic performance of Ag-deposited monophasic TiO_2_ (mainly anatase) under UV and/or visible irradiation has been attributed to surface plasmon resonance (SPR), with a resultant extension of the photoresponse of TiO_2_ in the visible-light region [39,40,41,42,43,44,45,46].

Photocatalysis terminates microbial cells in water. This finding supports the idea that the massive growth of cyanobacteria in eutrophic water can be prevented *a priori* by exploiting the photocatalytic activity of a catalyst. Despite extensive study in this field, to the best of the authors’ knowledge, the potential use of this method for the inactivation of cyanobacteria has not been widely explored. In this study, the photocatalytic inactivation of cyanobacteria in water are performed using a self-prepared, low-cost, non-toxic and mixed-phase Ag-deposited titanium dioxide (TiO_2_) that is coated on the surface of diatomite (DM) and used as a photocatalyst (Ag-TiO_2_/DM) with sunlight to inactivate cyanobacteria. The mutagenic activity of extracts of cyanobacterial extracellular polymeric substances (EPSs) is evaluated by the Ames test during the photocatalytic processes.

## 2. Materials and Methods

### 2.1. Preparation of Ag-TiO_2_ Film Supported on Diatomite

**Figure 1:**
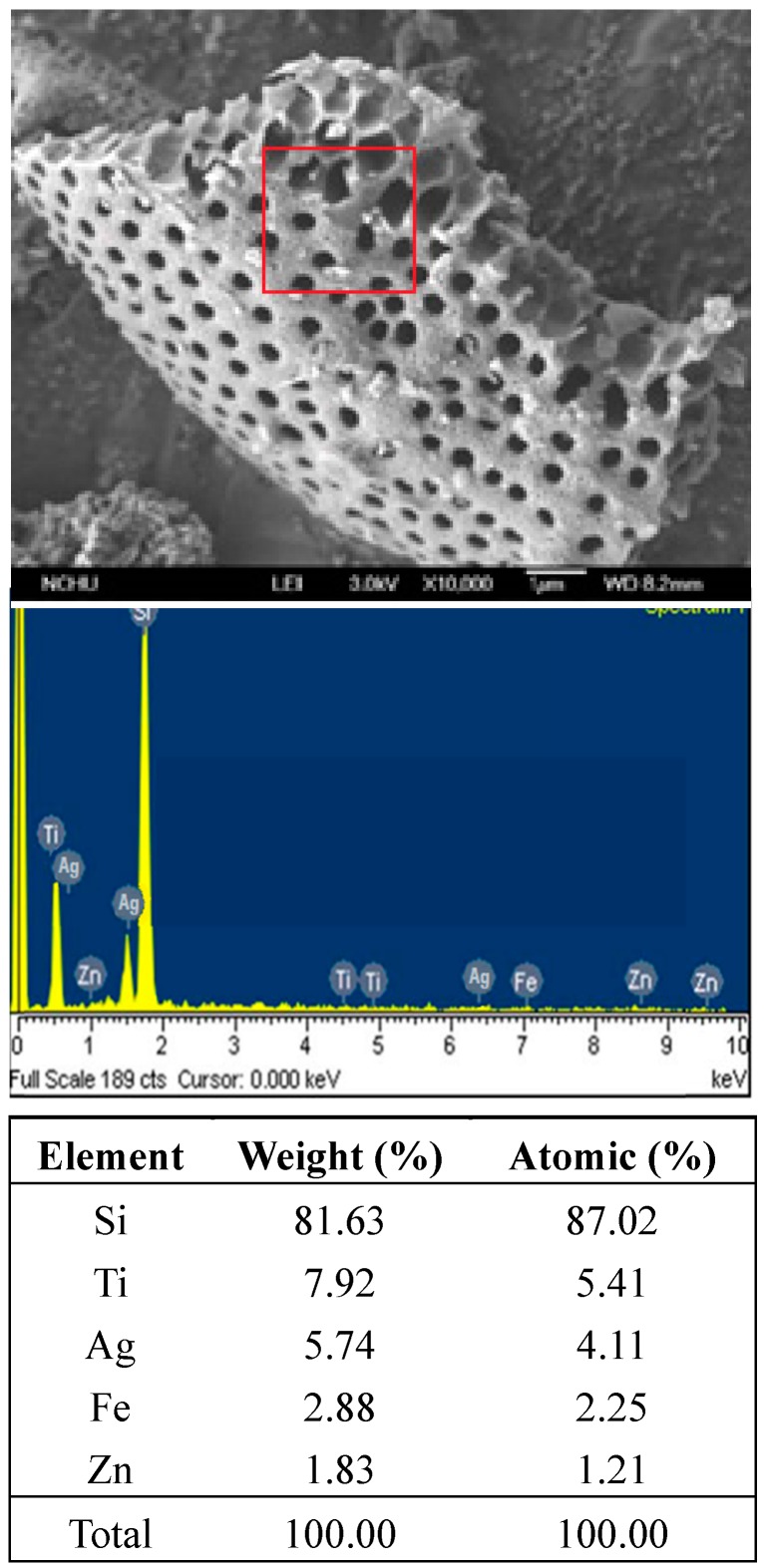
SEM images of Ag-TiO_2_ film on diatomite and EDAX analysis of Ag-TiO_2_/DM composite film.

Ag-deposited titania on the surface of diatomite was synthesized by the sol–gel method using titanium isopropoxide (Ti(OCH(CH_3_)_2_)_4_) purchased from Sigma–Aldrich (Sigma–Aldrich Co.: St. Louis, MO, USA) and isopropyl alcohol ((CH_3_)_2_CHOH) and silver nitrate (AgNO_3_) purchased from Merck (Merck KgaA: Darmstadt, Germany). Distilled water was used throughout the experiments. The monophasic titania sol was prepared by mixing 2.5 mm diatomite (5 g) with titanium isopropoxide and isopropyl alcohol in a 1:10 (*v*/*v*) ratio. A volume (0.5 mL) of distilled water was added dropwise with constant stirring for 24 h at room temperature at pH 5.0. The sol was then left to age for 12 h to form a gel before it was heated to 80 °C for 12 h. The resultant mixed-phase catalyst was then calcined at 500 °C for one hour to obtain the monophasic anatase. The Ag-deposited monophasic titania coated on the surface of diatomite was prepared by dropwise addition of an AgNO_3_ solution (1 weight %) into a mixture of 2.5 mm diatomite (5 g) in titanium isopropoxide and isopropyl alcohol (1:10 (*v*/*v*) ratio) with constant stirring. Subsequently, the sample was calcined at 500 °C with a ramping rate of 4 °C/min for one hour to yield an Ag-TiO_2_ film coated on diatomite (Ag-TiO_2_/DM, Figure 1). The thickness of the Ag-TiO_2_ film was determined to be 2.5 μm using stylus profilometry.

### 2.2. Cultivation of Cyanobacteria

Axenic cultures of the planktonic cyanobacteria *Microcystis aeruginosa* and *Oscillatoria tenuisa* were obtained from the Renyi-Tan Reservoir (Chiayi, Taiwan). Cyanobacterial samples were separated using a dissecting phase contrast microscope (model SSM-422L, Microtech, Hsinchu, Taiwan) and grown in standard culture media (DY-3 for *Microcystis aeruginosa* [47] and ASM-1 for *Oscillatoria tenuisa* [48]; Table 1).

**Table 1:** Concentrations of media nutrient during growth of cyanobacteria at 25 °C.

Cyanobacteria	Treatment	Nutrients			
		Light (Μe/m^2^s)	NO_3_^−1^-N (mg/L)	NH_3_-N (mg/L)	PO_4_^−3^-P (mg/L)
*Microcystis aeruginosa*	ASM-1 ^a^	30	32.8 ± 2.6	-	3.2 ± 0.3
*Oscillatoria tenuisa*	DY-3 ^b^	32	6.8 ± 0.4	-	0.7 ± 0.1

^a^ Source: cyanobacteria media adapted from Lehman [47]; ^b^ Source: cyanobacteria media adapted from Gorham *et al.* [48].

The pH was adjusted to 8.5 by buffering with bicine (*N,N*-2-bis(2-hydroxymethyl) glycine, C_6_H_13_NO_4_). Cultures were grown in a mineral liquid medium at 25 °C; air containing 5% (*v*/*v*) CO_2_ was bubbled through the cultures, which were continuously illuminated with cool white light and daylight from fluorescent lamps (30–33 Μe/m^2^ s) placed at the surfaces of the incubation chambers.

### 2.3. Photocatalytic Inactivation of Cyanobacteria

Photocatalytic reactions were performed in a semi-batch reactor (15 cm i.d., 60 cm height), which was illuminated from the outside by a solar lamp (Suntest AM1, Hanau, Germany) with a light spectral distribution in which approximately 0.5% of the emitted photons had wavelengths below 300 nm and approximately 7% had wavelengths between 300 and 400 nm. The emission spectrum between 400 and 800 nm was the solar spectrum.

The applied concentrations of the photocatalyst were maintained at 520−2100 mg/L Ag-TiO_2_/DM, corresponding to dosages of 50−200 mg TiO_2_/L and 36−148 mg Ag/L. Samples were collected at predetermined times (t) in the dark. Then, the solar lamp was turned on at an intensity of 1000 W/m^2^, and samples were collected at predetermined times for 24 h. Each experiment was conducted in triplicate.

The photosynthetic efficiency of cyanobacteria was determined using the carbon-14 method [49]. Samples were withdrawn from the reactor during the reaction at various reaction times and placed in light and dark bottles. A solution of radioactive carbonate (^14^CO_3_^−2^) was then added to the bottles. Following *in situ* incubation, the cyanobacteria were collected on a membrane filter and then treated with hydrochloric acid (HCl) fumes to remove inorganic carbon-14 before being assayed for radioactivity using a liquid scintillation counter (B2810TR, PerkinElmer: Norwalk, CT, USA). The quantity of carbon fixed by the cyanobacteria was proportional to the fraction of assimilated radioactive carbon.

### 2.4. Analytical Methods

Cyanobacterial cells were counted by flow cytometry, with chlorophyll-*a* fluorescence and cell viability as analytical parameters. After staining, aliquots of *Microcystis aeruginosa* and *Oscillatoria tenuisa* cultures were collected, centrifuged, and washed twice before being resuspended in buffered saline solution for analysis in a FACScan flow cytometer (D-48161 Partec, Münster, Germany) equipped with an argon-ion laser (blue light, 488 nm). Chlorophyll-*a* red fluorescence histograms of the particles that exhibited red fluorescence, which were clearly non-cyanobacterial particles, were used to set the gating levels. The fluorescence of cells stained with propidium iodide (PI) was measured to investigate cell viability. PI is a fluorescent dye that intercalates with double-stranded nucleic acids to generate red fluorescence when excited by blue light and cannot pass through intact cell membranes. However, when a cell dies, the membrane fails, allowing PI to enter and stain the nucleic acids. Accordingly, PI can be used to distinguish between live non-fluorescent cells and non-viable fluorescent cells.

In the staining procedure, aliquots of cells were washed as described above and stained with PI (Sigma: St. Louis, MO, USA) and SYBR green I (Sigma). A stock solution of 1 mM PI was prepared by dissolution in Milli-Q water and maintained at 4 °C. A stock solution of 1:100 (*v*/*v*) SYBR was prepared by adding Milli-Q water to a 1:10000 (*v*/*v*) commercial stock solution and maintained at −20 °C. One milliliter of cyanobacterial cells was stained by simultaneously adding 10 μL of PI and 10 μL of SYBR stock solution and incubating the resulting solution for 15 min at room temperature in the dark.

*M. aeruginosa* cells were counted and characterized using a FACScan flow cytometer. Cells were excited with an argon excitation laser (488 nm). SYBR green I fluorescence was detected in the FL1 detector (505–535 nm), and the data herein are expressed as the mean fluorescence intensity per cell (MFI). The fluorescence of PI was detected in the FL3 detector (565–605 nm), expressed as MFI. The flow cytometer was operated at a constant flow rate of 12 μL/min, and at least 5000 events were tested for every sample.

Chlorophyll-a was quantified using a spectrophotometric method [50]. The potassium ion (K^+^) concentrations in the filtrates were measured on an inductively coupled plasma-optical emission spectrometer (ICP-OES) (model 2100DV, Perkin-Elmer: Norwalk, CT, USA).

The formation potential of chlorination disinfection by-products (DBPs) was assessed following Standard Method 5710 [50]. The pH of the samples was maintained at 7.0 using a phosphate buffer solution. A stock chlorine solution was added, and all of the samples were stored in the dark at 25 °C. At the end of the seven-day incubation period, the samples contained a residual free chlorine concentration of 1–2 mg Cl_2_/L. Finally, a sodium sulfite solution was added to quench the residual chlorine. Trihalomethanes (THMs) were measured using a purge-and-trap gas chromatographic/mass spectrometric method on a GC/MS (model GC-6890N, MS-5973N, Agilent: Santa Clara, CA, USA) equipped with a purge-and-trap module (model LCS-2000, Tekmar: Cincinnati, OH, USA), consistent with Standard Method 6251 [50]. Six haloacetic acids (HAAs: monochloroacetic-, dichloroacetic-, trichloroacetic-, bromochloroacetic-, monobromoacetic- and dibromoacetic acid) were quantified by Standard Method 6232C [50] on a GC/ECD system (HP 6890N, Agilent: Santa Clara, CA, USA) equipped with a DB-1701 capillary column.

### 2.5. Mutagenicity Tests

The cyanobacterial suspensions were acidified with 2 N HCl to pH 2 and then concentrated on silica C_18_ cartridges (Sep-Pak Plus C_18_ cartridges, Waters Chromatography; Millipore: Darmstadt, Germany). The cartridges were activated before elution with 5 mL each of methanol and acetonitrile and washed with 100 mL of distilled water. Approximately 10 L of cyanobacterial suspension was adsorbed onto each cartridge, which was then dried under nitrogen gas (30 mL min^−1^). A similar procedure was utilized for the negative controls, with 10 L of mineral water obtained from an unpolluted ground source with an organic substance level (determined by quantifying dissolved organic carbon (DOC)) of 0.28 mg L^−1^ in place of the 10 L of culture.

After the solvent evaporated, the extracts were dissolved in dimethylsulfoxide (DMSO) and tested in duplicate at increasing concentrations (0.25, 0.5, 1, 2, and 4 of 1 equivalent volume per liter plate with a maximum volume of 200 μL of DMSO). *Salmonella typhimurium* TA 98 and TA 100 strains (supplied by the Development Center for Biotechnology: Taipei, Taiwan) with and without *in vitro* microsomal activation (S9 mix) [51] were used in the Ames test; TA 98 detects frame-shift mutagens, and TA 100 responds to base-pair substitution mutations. The positive controls were 2-nitrofluorene (Sigma) for TA98-S9 (10 μg per plate; 1011 ± 182 revertants per plate), sodium azide (Sigma) for TA100-S9 (10 μg per plate; 1226 ± 220 revertants per plate), and 2-aminofluorene (Sigma) for both strains with S9 (10 μg per plate; revertants per plate: 1487 ± 165 for TA98 + S9 and 1282 ± 178 for TA100 + S9). DMSO was tested as a negative control (100 μl per plate; revertants per plate: 9.4 ± 2.6 for TA98-S9, 43.8 ± 10.7 for TA100-S9, 17.3 ± 4.5 for TA98 + S9, and 45.4 ± 8.6 for TA100 + S9). The mean mutagenicity ratio from triplicate plate tests was determined. It is generally considered to be positive if two consecutive doses or the highest nontoxic dose is at least double that of the solvent control and at least two consecutive doses reveal a dose-response relationship [50].

## 3. Results and Discussion

### 3.1. Inhibition of Cyanobacterial Growth

**Figure 2:**
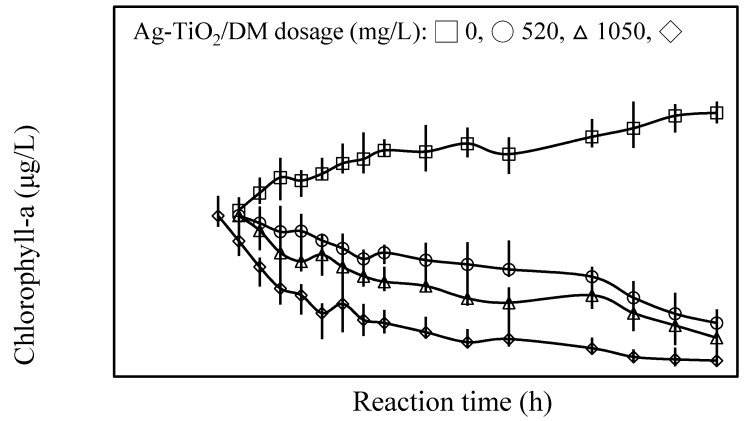
The concentrations of chlorophyll-a as a function of photocatalytic reaction time in *Microcystis aeruginosa* suspensions.

**Figure 3:**
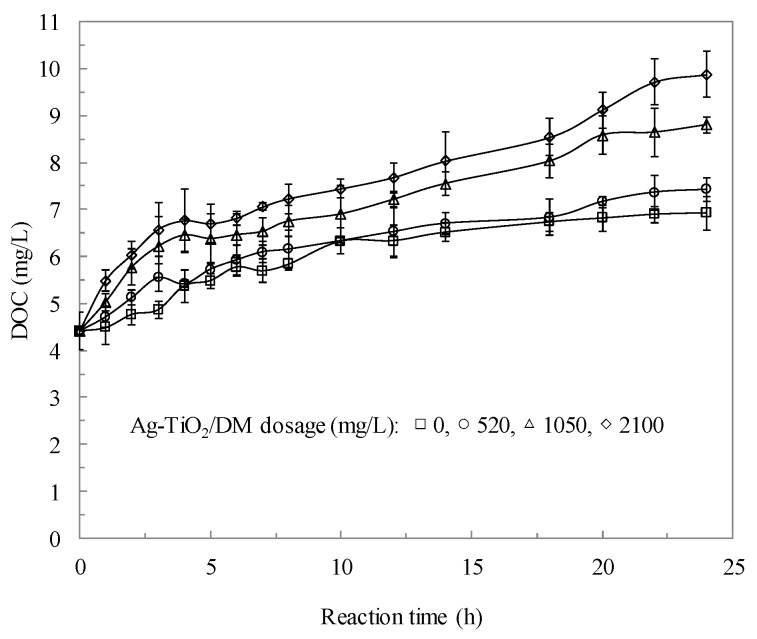
The concentrations of DOC as a function of photocatalytic reaction time in *Microcystis aeruginosa* suspensions.

The properties of axenic cultures of planktonic cyanobacterial suspensions were determined before and after the photocatalytic reaction to determine how photocatalysis influences the inhibition of cyanobacterial growth. Experimentally, a *Microcystis aeruginosa* suspension with 2.5 × 10^5^ cells/mL was photocatalyzed with 520−2100 mg/L Ag-TiO_2_/DM, corresponding to dosages of 50−200 mg TiO_2_/L and 36−148 mg Ag/L. Photocatalysis with higher Ag-TiO_2_ dosages resulted in a 17%−92% lower chlorophyll content in the cyanobacterial suspension (Figure 2). The photocatalyzed process was accompanied by an increase in the DOC content of the suspension by 64%−115% (Figure 3). Normal exposure to sunlight did not influence algal chlorophyll pigments as expected [52]. Algal cells contain a large quantity of green pigment in the chloroplasts of the cells. The loss of chlorophyll is attributed to the degradation of algal pigment components such as chlorophyll-*a*, chlorophyll-*b*, and phaeophytin-*a* by Ag-TiO_2_-promoted oxidation. The apparent change in color of photocatalyzed algal samples from blue-green to brown is caused by a damaging oxidative process at the Ag-TiO_2_ surfaces that eventually degrades the chlorophylls and other pigments, which is consistent with the data on TiO_2_-mediated photocatalysis obtained by Hong and Otaki [53].

Photocatalyzed samples were placed in cylinders and maintained at 25 °C under a fluorescent lamp in an incubation chamber with a 12 h/12 h light/dark cycle. Samples were periodically withdrawn for microscopic observation and accounting.

**Figure 4:**
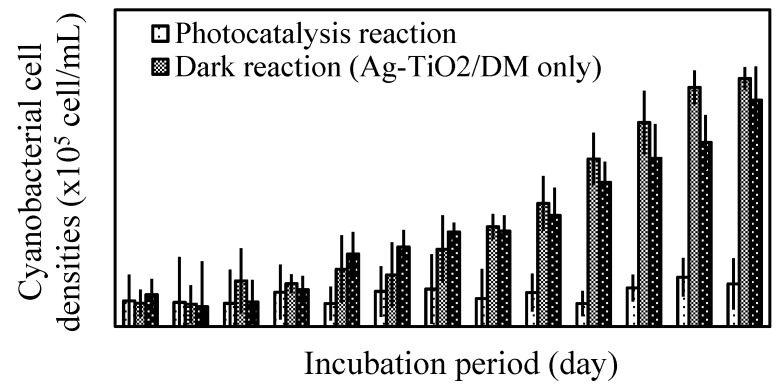
Time courses of cell densities of *Microcystis aeruginosa* cultures during incubaction period.

According to Figure 4, only slight changes in the cyanobacterial cell density were observed after photocatalysis. However, after the fourth day of incubation under fluorescent light, the photocatalyzed and control samples (without photocatalysis) significantly differed in cell density. By the end of the 12th day, the cell densities in all photocatalyzed samples were decreased 92% compared to the densities of the control group.

After the fourth day, the green color of the cyanobacterial cells had gradually faded, and, by the end of the 12th day, either all of these cells had completely faded or their cell walls had broken. However, the cells to which 2100 mg/L Ag-TiO_2_/DM was added without light illumination (dark reaction) continued to survive. The cell densities in these samples remained the same throughout the incubation period, whereas the cell density in the control samples increased from (3.2 ± 1.9) × 10^5^ to (6.3 ± 2.8) × 10^7^ cells/mL. Additionally, the sample with only photocatalyst Ag-TiO_2_/DM (without light excitation) increased from (1.7 ± 0.6) × 10^5^ to (8.9 ± 3.3) × 10^7^ cells/mL. Notably, the non-photolyzed samples and the control group differed only slightly in cell density after 12 days of incubation. These variations in cell density and morphology indicate that the increase in DOC content in photocatalyzed samples is caused by either an increase in the liberation of extracellular organic matter or the release of lysate from lysed cyanobacterial cells.

**Figure 5:**
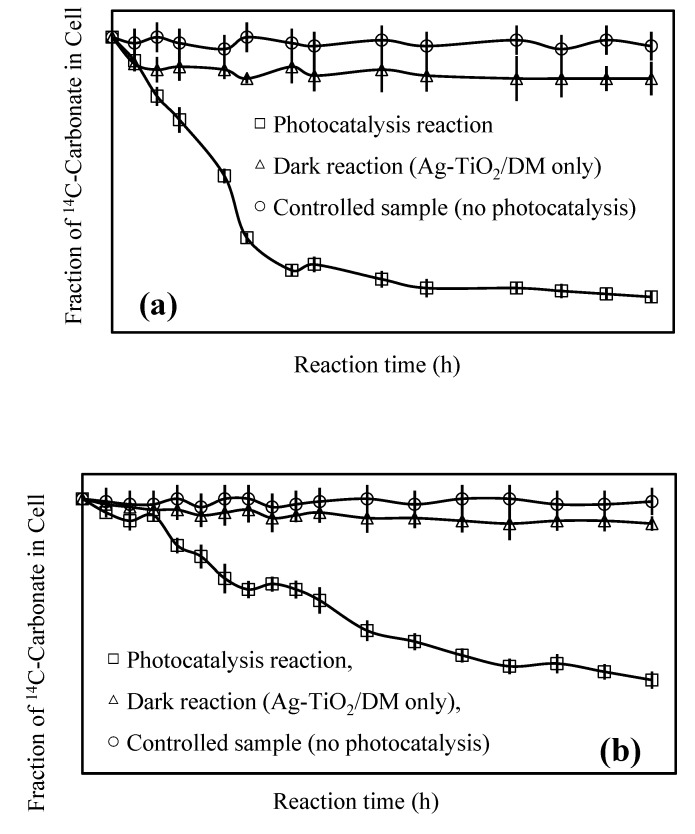
Relative changes in photosynthetic activity of *Oscillateria tenuisa* (**a**) and *Microcystis aeruginosa* (**b**) during photocatalytic reaction.

Figure 5 presents the relative changes in the photosynthetic activities of *Microcystis aeruginosa* and *Oscillatoria tenuisa* during the photocatalytic reaction. The relative ^14^C assimilation, recorded on the *y*-axis, refers to the ratio of the ^14^C-assimilation before the reaction to that during the reaction [54,55]. Most of the inactivation of the photosynthetic activity of *Oscillatoria tenuisa* occurred within 12 h. However, in the case of *Microcystis aeruginosa*, the photocatalytic inactivation efficiency was significantly lower. Approximately 55% of the cells lost their photosynthetic activity within 24 h. We believe that *Microcystis aeruginosa* exhibits lower inactivation because of the presence of extracellular polymeric secretions that surround the cells. EPSs with a high molecular weight must have prevented the oxidation of the cyanobacterial cells through contact with the Ag-TiO_2_ photocatalyst.

**Figure 6:**
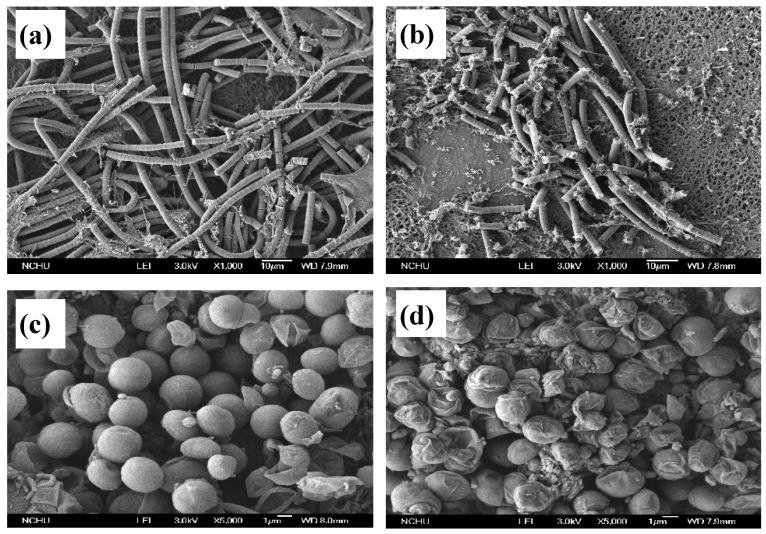
Electron micrographs of cyanobacteria: *Oscillateria tenuisa* (**a**) original (control sample); (**b**) after photocatalytic reaction; *Microcystis aeruginosa* (**c**) original (control sample), and (**d**) after photocatalytic reaction.

The SEM microscopic images of the two cyanobacteria *Microcystis aeruginosa* and *Oscillatoria tenuisa*, before and after 24 h of the photocatalyzed reaction (Ag-TiO_2_/DM dosage = 2100 mg/L) significantly differed from each other (Figure 6). *Oscillatoria tenuisa* is a typical filamentous cyanobacterium with cells that have scattered solitary granules and rounded apical cells; its trichomes are straight, unconstricted at cross-walls, and cylindrical. When the original *Oscillatoria tenuisa* cells underwent a photocatalyzed reaction for approximately 10 h, the cylindrical skeleton of *Oscillatoria tenuisa* broke. *Microcystis aeruginosa* is a typical nonfilamentous and colonial cyanobacterium [56]. The colonies of *Microcystis aeruginosa* consist of hundreds of spherical cells in a mucilaginous sheath. The spherical cell colonies were completely isolated following the photocatalyzed reaction. Moreover, the extent of microorganism death due to the photocatalyzed reaction was inversely proportional to the thickness and complexity of the cell wall [57]. The difference between the sensitivities of *Microcystis aeruginosa* and *Oscillatoria tenuisa* to the photocatalytic reactions is one of the main reasons for the observed differences in inactivation efficiency.

**Figure 7:**
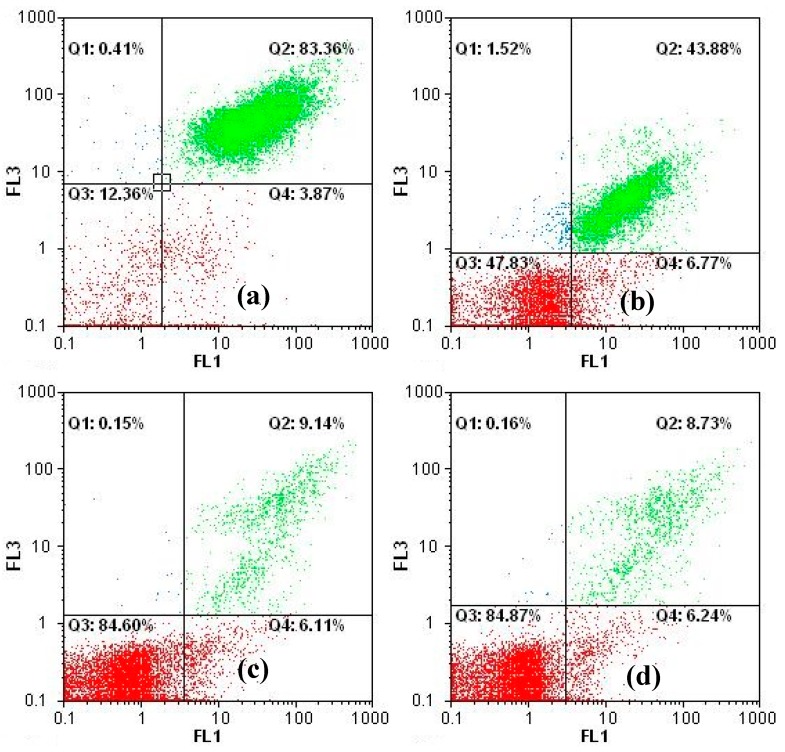
Flow cytometry images of *Microcystis aeruginosa* cells by FL1 (505–535 nm) and FL3 (565–605 nm) detectors. Ag-TiO_2_/DM dosage (mg/L): (**a**) 0, (**b**) 520, (**c**) 1050, and (**d**) 2100.

Figure 7 displays a flow cytometric image of *Microcystis aeruginosa* cells that have been damaged by the photocatalytic reaction. As the dosage of Ag-TiO_2_/DM increased (Figure 7a–d), the numbers of vegetative cells in the square region decreased, and most dots therein (fragments of cells or dead cells that exhibited less chlorophyll-*a* fluorescence than the vegetative cells) appeared in the bottom-left. The percentage of dead cells increased rapidly to 12.36% and 84.87% when the concentration of Ag-TiO_2_/DM was 0 mg/L and 2100 mg/L, respectively.

### 3.2. Fate of Metabolic Products During Photocatalysis

**Figure 8:**
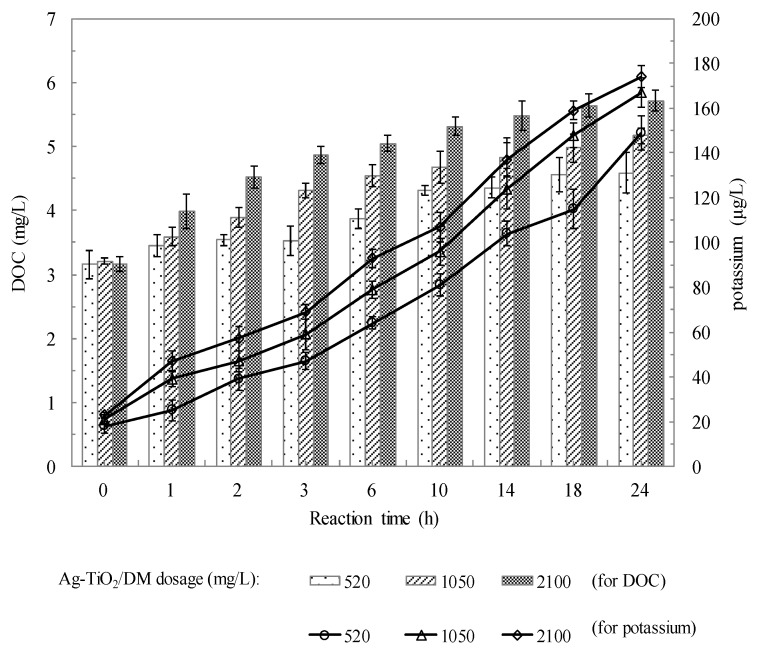
The concentrations of DOC and potassium ion as a function of photocatalytic reaction time.

The bulk properties of the *Microcystis aeruginosa* suspension were determined before and after the photocatalyzed reaction to understand how photocatalysis affects the characteristics of the DOC suspension. According to Figure 8, a suspension with a concentration of 2.57 × 10^5^ cells/mL was photocatalyzed at pH 7.5 with an Ag-TiO_2_/DM dosage of 520–2100 mg/L and a reaction time of 24 h.

The DOC concentration increased with the reaction time from 0 to 24 h. Larger quantities of DOC were observed at higher Ag-TiO_2_/DM dosages. The above results reveal that the increase in the DOC suspension was caused by the increased liberation of extracellular organic matter and cell-wall polysaccharides during photocatalysis. Figure 8 also plots the time-dose-response curves, which relate to the cell wall damage, obtained by quantifying the cellular release of K^+^. The release of K^+^ by cyanobacterial water increased steadily with the Ag-TiO_2_/DM dosage. The degree of K^+^ leakage is likely related to the degree of damage to the cell wall and can thus be used as a physiological indicator of toxic effects [58,59].

**Figure 9:**
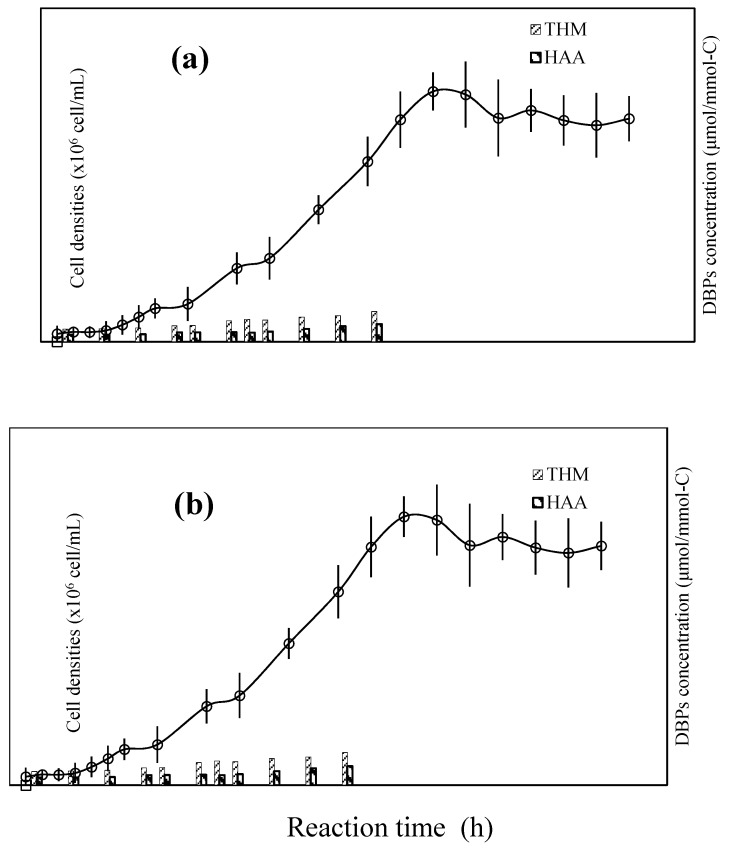
Formation potential of DBPs during all growth phases of cyanobacteria cultures. (**a**) *Microcystis aeruginosa*, (**b**) *Oscillateria tenuisa*.

The contribution of EPSs to the formation of DBPs during chlorination was investigated. Figure 9 shows that the total THM formation potential (THMFP, comprising the four THM species) and total HAA formation potential (HAAFP, comprising the six HAA species) yields of cyanobacterial EPSs varied with the duration of growth after photocatalysis treatment. The EPSs of *Microcystis aeruginosa* and *Oscillatoria tenuisa* exhibited a mild potential for DBP formation. The yields of total THMs (TTHMs) and total HAAs (THAAs) were closely related to the growth phase. During the lag phase, both the TTHM and the THAA yields remained constant and slightly increased at the beginning of the exponential phase. The yields fluctuated at the end of the exponential phase and then steadily increased in the stationary phase. The specific DBP yields (yield/DOC) of *Microcystis aeruginosa* were 3.42–8.15 μmol/mmol C for TTHMs and 1.90–4.76 μmol/mmol-C for THAAs, and those of *Oscillatoria tenuisa* were slightly higher.

Table 2 shows the data on the number of revertant bacteria of the TA-98 and TA-100 tester strains with and without activation after treatment with photocatalysis water sample concentrates. The cyanobacterial EPS extracts exhibited negative mutagenic activity with the TA-98 tester strain with and without activation. The same extracts showed positive mutagenic activity with the TA-100 tester strain, but only after activation following extraction. According to the results herein, the mutagenic effect for the TA-100 tester strain with metabolic activation of test extracts was dominated by the increased amount of suspended EPSs during photocatalysis likely due to the liberation of extracellular organic and cell-wall polysaccharides.

**Table 2:** Mutagenic activity of cyanobacterial EPS extracts in TA98 and TA100 *Salmonella* strains.

Samples	Dose (L Per Plate)	Mean Revertant Colonies/Plate						
		TA98					TA100	
		−S9 ^a^		+S9 ^b^		−S9		+S9
Blank control	0	27 ± 7 ^c^		51 ± 6		184 ± 15		207 ± 24
*Microcystis aeruginosa*	4.0	52 ± 8		92 ± 12		322 ± 37		452 ± 30 ^d^
	2.0	42 ± 4		76 ± 16		274 ± 25		436 ± 19 ^d^
	1.0	37 ± 6		68 ± 8		245 ± 13		365 ± 27
	0.5	33 ± 7		66 ± 5		196 ± 18		377 ± 33
	0.25	31 ± 5		60 ± 7		208 ± 26		349 ± 25
*Oscillatoria tenuisa*	4.0	45 ± 3		86 ± 11		280 ± 16		428 ± 35 ^d^
	2.0	48 ± 6		79 ± 12		286 ± 28		386 ± 21
	1.0	37 ± 5		78 ± 5		256 ± 12		391 ± 28
	0.5	31 ± 6		62 ± 4		252 ± 8		345 ± 52
	0.25	34 ± 7		55 ± 13		197 ± 15		293 ± 18

^a^ Test strains without S9. ^b^ Test strains with S9. ^c^ Data are the mean of triplicate plates. ^d^ Mutagenicity ratio (revertants for samples)/(revertants for blank control) > 2.

## 4. Conclusions

The above observations indicate that processes photocatalyzed by hybrid Ag-TiO_2_/DM significantly inhibit the photosynthetic activity of cyanobacteria under sunlight. Inactivation experiments were performed on two species of cyanobacteria: *Microcystis aeruginosa* and *Oscillatoria tenuisa*. Irradiation by sunlight in the presence of the Ag-TiO_2_/DM catalyst greatly altered the cyanobacterial structure. The cylindrical skeleton of *Oscillatoria tenuisa* broke, and most of the isolated cells ceased their photo- synthetic activity. Although colonies of *Microcystis aeruginosa* cells were completely separated into individual spherical cells, the inactivation efficiency of *Microcystis aeruginosa* was somewhat lower than that of *Oscillatoria tenuisa*. We believe that the lower efficiency is due to EPSs surrounding the cells of *Microcystis aeruginosa*. Furthermore, cyanobacterial cells released organic substances during the photocatalyzed reaction. A high dosage of Ag-TiO_2_/DM catalyst significantly increased the DOC concentration of the cyanobacterial suspension, apparently by increasing the liberation of EPSs. Analysis of the potential for DPS formation and the Ames test of the liberation of EPSs indicated that photocatalysis may slightly increase the DBP and mutagenic potential.
